# Facile synthesis of nanostructured cobalt pigments by Co- A zeolite thermal conversion and its application in porcelain manufacture

**DOI:** 10.1038/s41598-020-67282-1

**Published:** 2020-06-23

**Authors:** Assunta Campanile, Barbara Liguori, Ottavio Marino, Gennaro Cavaliere, Valter Luca De Bartolomeis, Domenico Caputo

**Affiliations:** 1 ACLabs – Dipartimento di Ingegneria Chimica, dei Materiali e della Produzione Industriale Università di Napoli Federico II, P.le V. Tecchio 80, 80125 Napoli, Italia; 2 INSTM - Consorzio Interuniversitario Nazionale per la Scienza e Tecnologia dei Materiali, Via G. Giusti 9, 50121 Firenze, Italia; 3 Istituto di Istruzione Superiore ad Indirizzo Raro “Caselli - De Sanctis” di Napoli, Parco di Capodimonte, 80131 Napoli, Italia

**Keywords:** Synthesis and processing, Structure of solids and liquids

## Abstract

An innovative and facile synthesis of cobalt based inorganic pigment was carried out by means of a low energy thermal conversion of a cobalt-exchanged zeolite. The Na-A [LTA] zeolite was used, since it is a low cost and easily available raw material. The ion exchange mechanism allowed to control, at microscopic level, the composition of the zeolitic precursor. Several chromatic effects could be obtained by varying the treatment temperature and/or the cobalt concentration in the contact solution. The reliability of these new zeolite-based pigments was tested in porcelain manufacture, in collaboration with the Institute for the *Capodimonte porcelain* “Giovanni Caselli”. The developed cobalt pigments were successfully tested either in the porcelain mixture to obtain a coloured product or in the decoration step (such as *ingobbio*, colored glazes and “third fire decoration”).

## Introduction

The aesthetic appearance, especially the colour, is the major factor that influences the pleasure degree of a ceramic product. The use of pigments is among the most effective methods to definitively colour a ceramic support. Natural pigments are available in nature and they have been obtained since prehistoric times from natural minerals, consequently they contain a lot of impurities and are hardly reproducible^1^. On the other hand, the synthetic ones are pure, uniform and stable, both chemically and thermally, but they are more expensive than the firsts, because of the higher cost of the raw materials (very pure metallic compounds and expensive additive to give specific properties) and synthesis process (fine grinding, mixing, heat treatment at high temperatures, i.e. 1400 °C). In order to be used in coloured ceramic products inorganic pigments have to be thermally stable at the firing temperature and toward the actions of molten glasses (frits and/or sintering aids)^2,3^. Blue pigments have been widely used from early age for surface decoration of stylistically different classes of pottery, and also in the bulk coloration of polished unglazed ceramics.

The traditional source of blue in currently known ceramic pigments contains cobalt ion as the chromophore^4–9^. The most common blue pigments used in ceramic industry are based on cobalt oxide or cobalt aluminate. Several methods are known for obtaining cobalt aluminate such as hydrothermal reactions, complexation or solid-state reactions, coprecipitation, sol-gel methods, combustion synthesis^6,10–12^. Peymannia *et al*.^13^ proposed the synthesis of CoAl_2_O_4_ nanostructured pigments by an electrostatic or steric stabilization method. The additives used to preserve the nanoscale size of the pigments make this method less competitive in economic terms. An alternative method is based on covering alumina nanoparticles with cobalt by a calcination treatment at 850–1000 °C^14^.

One of the most interesting applications of zeolites is the use in the original cation form or after suitable pre-exchange, as precursors for the production of ceramics^15–19^. Firing of zeolitic powders results in the breakdown of the zeolite structure and, depending on its chemical composition, in its conversion into amorphous or crystalline ceramic materials. All the advantages of this ceramic manufacture procedure comparing with traditional methods such as firing of oxides, have been deeply discussed in literature^16,17^. Introducing transition metal cations into zeolite structure gives rise to a distinctive coloration of the zeolitic exchanger, which can be preserve upon heating. So it is possible to directly obtain coloured ceramics or also powders, which may behave as ceramic pigments^20^. This procedure allows a homogeneous distribution of the extra-framework cations in zeolites, resulting in a uniform distribution of colouring centres within the structure with a consequent more uniform and safe colouring of the ceramic product after thermal treatment.

In the present paper sodium zeolite A [LTA] has been selected as white precursor and cobalt ion has been chosen as chromophore agent. An innovative and facile synthesis of a Co based pigment is proposed based on a low energy thermal transformation of the cobalt exchanged zeolitic precursor. Firstly, chemical and thermal characterization of the zeolite based blue pigments are reported and discussed.

The reliability of these pigments in porcelain manufacture was tested in a research activity developed at the Federico II University of Napoli (Italy) in collaboration with Institute for the Capodimonte porcelain “Giovanni Caselli”. The new cobalt pigments were firstly tested in the porcelain mixture to obtain a coloured product. Then the efficacy, in term of colour and aesthetic effect, was tested in some of the most common decoration methods such as *ingobbio*, colored glazes and “third fire decoration”.

## Preparation and characterization of the pigment

### Materials and Methods

The synthetic zeolite is a LTA type in sodic form supplied by Sasol company (Italy). This choice is due to the multivalent cation selectivity of zeolite A^16,17^, its low cost and wide availability on the market. The cation exchange capacity calculated on its ideal formula (NaAlSiO_4_·2.25·H_2_O) is 5.48 meq/g. The granulometry of the zeolite sample is between 0.5 and 5 µm.

Cobalt was chosen as chromophore agent. Cobalt solutions were prepared by dissolving appropriate quantities of cobalt nitrate, Co (NO_3_) _2_ · 6H_2_O, (supplied by Baker) in distilled water.

The pigment precursors were prepared by loading zeolite A with different Co^2+^ contents. For this purpose, suitable quantities of zeolite were kept in contact, under continuous stirring for 24 hours, with fixed volumes of cobalt solution at a concentration equal to 0.05, 0.1 or 0.15 M. For all the samples a solid to liquid ratio of 50 g/l was used.

The chemical composition was obtained by the following procedure: known amounts of each sample were previously calcined at 900 °C and then subjected to digestion in a microwave furnace (Perkin-Elmer Multiwave 3000 furnace) in a standard acid solution, prepared by mixing 1 ml of HCl (37%, w / w), 1 ml of HNO_3_ (65%, w / w) and 4 ml of HF (39.5%, w / w). After the addition of 24 ml of an 8 M H_3_BO_3_ solution for the fluoride’s complexation, the obtained solution was analysed with an atomic emission spectrophotometer equipped with electromagnetic induction plasma (ICP-OES, Perkin Helmer).

In order to obtain the Co-pigments, the resulting powders were treated at different temperatures from 600 to 1100 °C for one hour with heating rate of 10 °C/min.

The nature of the crystalline phases was evaluated by X-ray diffractometry (X’Pert Pro diffractometer, PANalytical) with the following test conditions: Cu Kα radiation, 2θ interval: 5–60° range, step 0.02° 2θ; 0.02° 2θ / s; scan speed; slide opening 0.5.

Co-A samples were subjected to simultaneous differential thermal analysis (DTA) and thermogravimetry (TG), using a Netzsch thermoanalyzer model STA 409 Luxx (weight of the sample: 10 mg; heating rate: 10 °C min−1; reference material: α-Al_2_O_3_; atmosphere: N_2_).

The colour of a pigment is usually defined using the CIELAB method, which measures the absorption intensity in the visible region at three fixed wavelengths, related to the absorption intensity values of a white standard (BaCO_3_) and mathematically processed, allow to obtain three parameters, L*, a* and b*, that define Hunter’s colour space. These parameters define the gloss and the predominance of the red/green components (from + a * to -a *) and yellow/blue, respectively (from + b * to -b *). By plotting these three parameters in graph, the chromatic diagram is obtained. The three colorimetric coordinates (L*, a* and b*) were measured with the colorimeter Micro Color Data Station Dr Lange.

The thermal stability of the cobalt pigment was evaluated in the firing temperature range typical of a porcelain mixture (1240 and 1280 °C for oxidizing or reducing atmosphere in the furnace, respectively)^21^.

### Results

The results of the chemical analyses showed that when the concentration of Co in the solution increases, the Co / Na ratio is included between 0.7 to 2.1 (Table 1). However, while passing from a concentration of 0.05 to 0.1 M it is possible to obtain a more than doubled ratio, the further increase to 0.15 M leads to an increase in the ratio of less than 20%. The cation exchange allowed to control the composition of the precursors at the atomic level^19,20,22^. In fact, Riley and Seff^22^ studying the crystal structure of a partially cobalt exchanged zeolite A, have demonstrated that after the cation exchange, the cobalt ions are located inside the zeolitic framework in two distinct crystallographic sites. Previous results^15–17^ about the suitability of cation-exchanged zeolites as precursors for ceramic materials have demonstrated that selecting the right zeolite type in terms of chemical composition, and subjecting it to exchange with the right cation, it was possible to reproduce the stoichiometric composition of a series of aluminosilicates. Then a suitable heat treatment resulted in (a) dehydration of the crystalline zeolite structure; (b) irreversible structural collapse with the formation of an amorphous phase and (c) crystallization of the corresponding aluminosilicate from the amorphous phase.

**Table 1:** Cobalt content in samples obtained with different nitrate solutions.

Sample		Co			Na		Co/Na
	ppm	mg/g	meq/g	ppm	mg/g	meq/g	
0.05-A	378.9	63.2	2.1	528.1	88.0	3.8	0.7
0.10-A	604.8	100.8	3.4	360.9	60.1	2.6	1.7
0.15-A	610.6	101.8	3.5	296.2	49.4	2.1	2.1

When a sodic zeolite A is heat treated, due to its good thermal stability, the structure remains up to about 850 °C, then collapse begins with simultaneous recrystallization in a mixture of nepheline and carnegieite (aluminium-silicates belonging to the feldspathoid family)^16,17^. Nevertheless, the thermal stability of a zeolite strongly depends on its cationic composition. In fact, during the heat treatment, due compensating the loss of some oxygen atoms of water molecules with reticular ones, extra-reticular cations move towards new equilibrium positions, causing a distortion of the structure, which can evolve towards the breaking of zeolitic framework and following possible recrystallization^16,20^.

**Figure 1:**
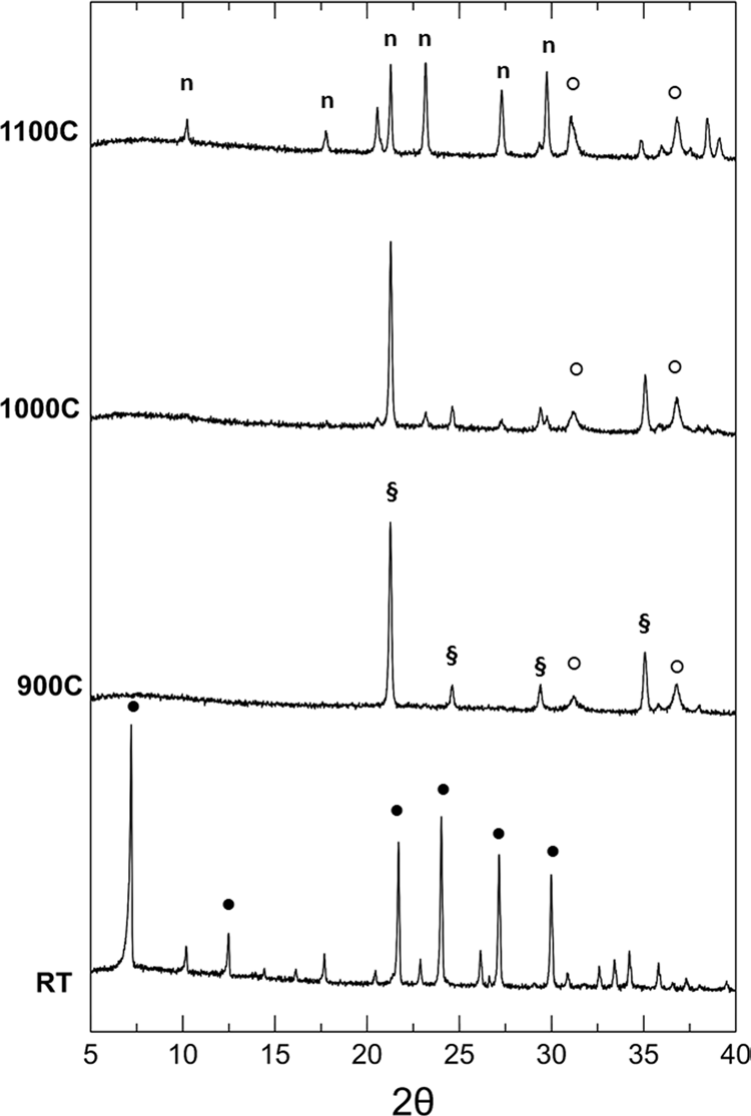
Thermal evolution of the crystalline phases in the 0.05-A sample (●: Co/Na-A; § =sodium aluminate; ○: cobalt aluminate; n = nepheline).

Following the thermal evolution of the cobalt exchanged zeolite at different cation contents, it is possible to observe a recrystallization in nepheline and / or carnegieite (determined by the presence of the sodium in the starting A zeolite) and in cobalt aluminate (CoAl_2_O_4_) if the Co/Na ratio is less than 1 (Fig. 1). In particular, for the 0.05-A sample the Co- exchange did not provide a significant distortion of the zeolite framework, although a shift in the main diffraction peaks can be detected (Fig. 1). Following the 900 °C treatment, the zeolitic structure is completely collapsed and recrystallizes in the sodium silico aluminate (NaAlSiO_4_). Up to 1000 °C the sodium silicate aluminate is present in the form of carnegieite (ICCD PDF # 52–1342), while at 1100 °C it turns into nepheline (ICCD PDF # 79-0993). The presence of cobalt, although in small quantities, also promotes the crystallization of another crystalline phase: cobalt aluminate (CoAl_2_O_4_), already present starting from 900 °C. The higher Na content in the system promotes the crystallization of sodium silico-aluminates compared to the cobalt aluminate, which crystallizes in a small percentage.

**Figure 2:**
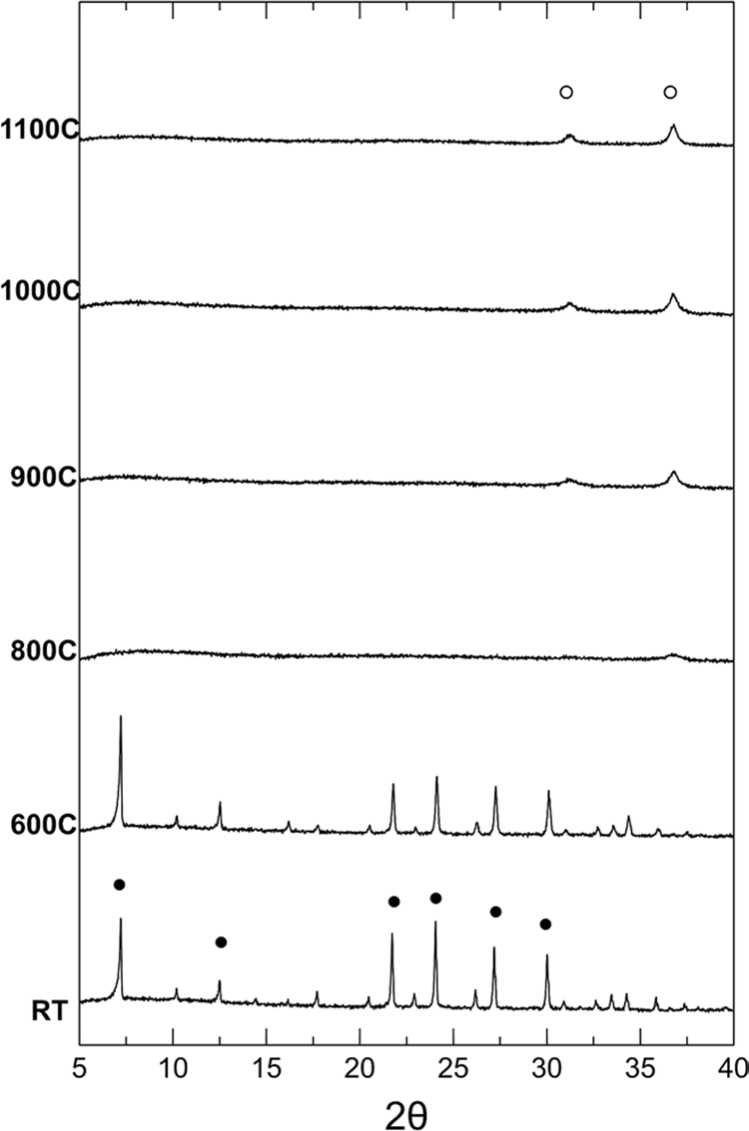
Thermal evolution of the crystalline phases in the 0.1-A sample (●: Co/Na-A; ○: cobalt aluminate).

The 0.1-A sample has an analogous crystalline structure to the 0.05-A sample (Fig. 2). However, the presence of a greater quantity of cobalt (about 100 mg per g of zeolite) is responsible for a significant change in the thermal evolution of the phases: the greater instability of the lattice together with a higher Co / Na ratio, inhibits the growth of silicate aluminate sodium phases, promoting the growth of cobalt aluminate. This phase, in fact, is the only crystalline phase already present at 800 °C together with an amorphous phase, rich in sodium, as shown by the band present at low angles. A further increase in temperature does not produce substantial changes in the crystalline phases present in the samples treated at 900, 1000 and 1100 °C.

Similar results (data reported in supporting information) have been obtained with the 0.15-A sample due to a very similar Co / Na ratio (1.7 and 2.1 respectively).

**Figure 3:**
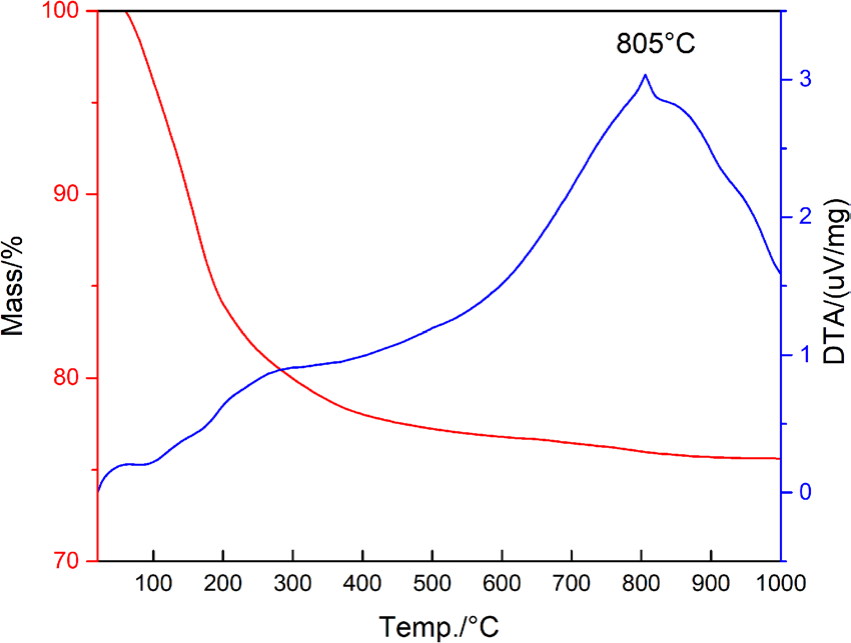
TG/DTA curve of the 01-A sample.

The thermal behaviour of 0.1-A is shown by the DTA/TG curves in Fig. 3. The DTA curve shows some endothermic effects at lower temperatures connected to zeolite dehydration upon heating. The thermogram presents also an exothermic peak at about 800 °C connected to the crystallization of cobalt aluminate from the amorphous phase, as demonstrated by the XRD pattern of the material, treated at that temperature and then air quenched (see Fig. 2).

**Figure 4:**
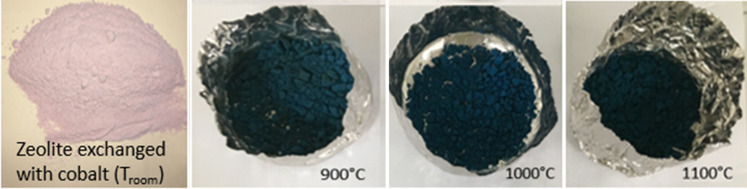
Colour of the powders of sodium Zeolite A, Co-exchanged A (01-A) and after thermal treatments.

The powders exchanged with cobalt, which are light pink at room temperature (pink is, in fact, the colour of the Co^2 +^ hydrate ion), take on a blue colour following the heat treatment from temperatures higher than 800 °C (Fig. 4).

**Figure 5:**
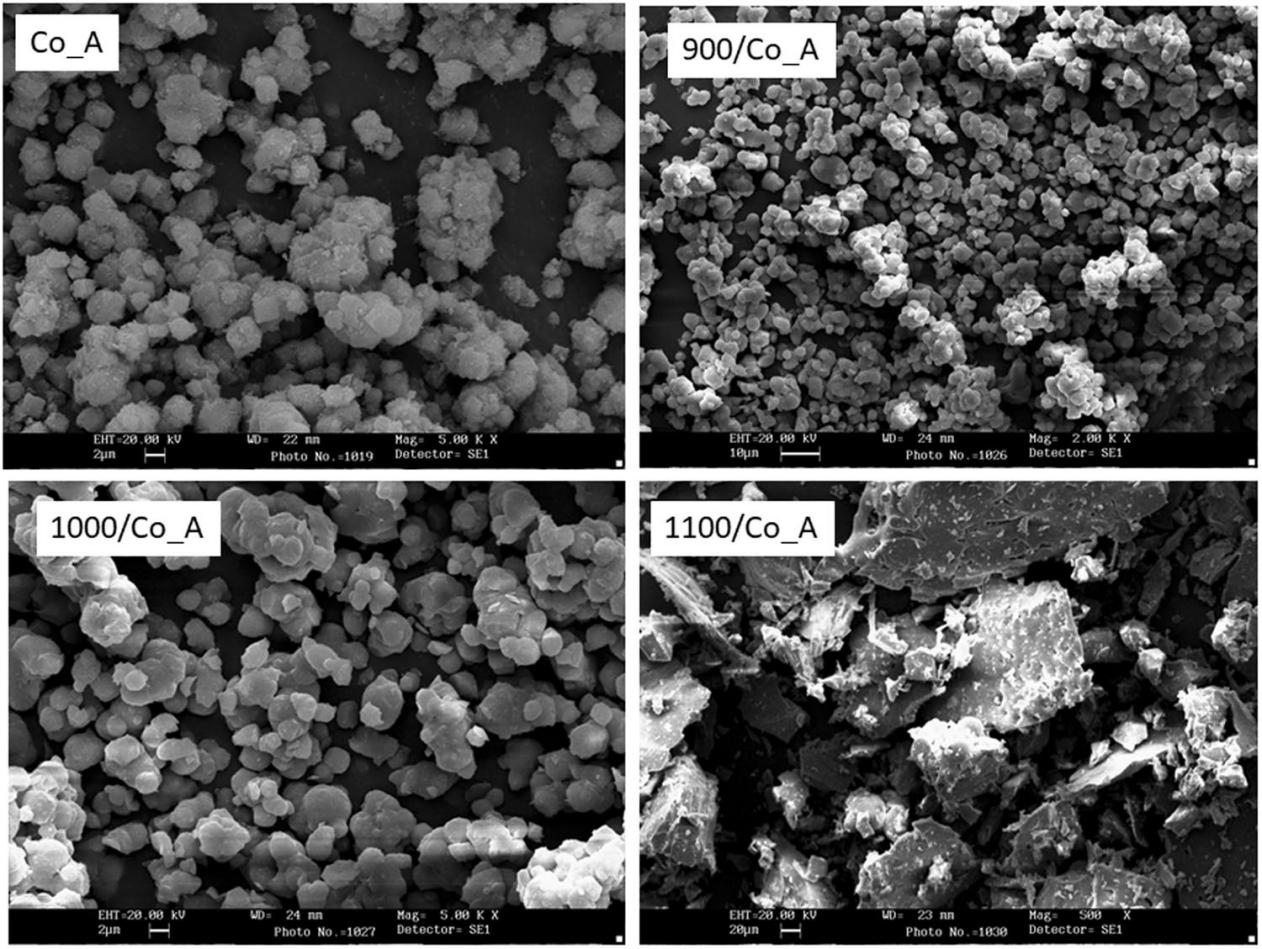
Morphological aspect of the pigments at different heat-treatments.

Figure 5 shows SEM morphologies of the Co-exchanged zeolitic powders untreated and after each thermal treatment. The thermal treatment at 900 °C did not affect the shape and dimension of the particles, which appeared similar to the raw zeolitic powders (Fig. 5). Nevertheless, increasing the temperature up to 1000 °C induced a partial sinterization, which can involve an improvement in the hardness of the pigment powders.

Since the optimum colouring efficiency can be obtained with a particle size of the powdered ceramic pigment in the range (1–10 µm)^23^, the 900 °C treated samples were selected for the colorimetric analysis and compared with three typical blue pigments of ceramic industry (Table 2). Inspecting data in Table 2, L* represents the brightness of the ceramic pigment, b* indicates the blue colour with a high negative value representing substantial blue intensity, and a* shows the green colour with a positive value indicating red and negative value representing green. The L* appears substantially independent from the Co amount in zeolite, whereas the a* change from a negative to a positive value with increasing Co content. The b* was the lowest for the 0.1-A indicating that the ceramic pigment obtained is the bluest.

**Table 2:** Colorimetric coordinates of coloured mixtures 1.

Pigments		Coordinates		Rendered Colour*
	L*	a*	b*	
0.05-A/900 °C	47.9	−1.3	−21.1	
0.10-A/900 °C	41.9	1.1	−24.8	
0.15-A/900 °C	36.7	1.2	−21.2	
Sevres Blue	48.0	1.6	−22.9	
Ultramarine blue	50.5	−11.5	−14.5	
Cobalt blue	42.8	1.5	−30.1	
CoAl_2_O_4_ [13]	48.1	−19.3	−31.1	
Cobalt aluminate nanoparticles [14]	36.1	−9.04	−36.63	

*the rendered colours were obtained by *Nixsensor color-converter* by colorimetric coordinates.

## Use of the zeolite based cobalt pigment in porcelain

Following the above reported results, the 0.1-A sample treated at 900 °C was selected and the blue pigment was tested in porcelain manufacture.

The cobalt pigments were firstly used in the porcelain mixture to obtain a coloured product.

**Figure 6:**
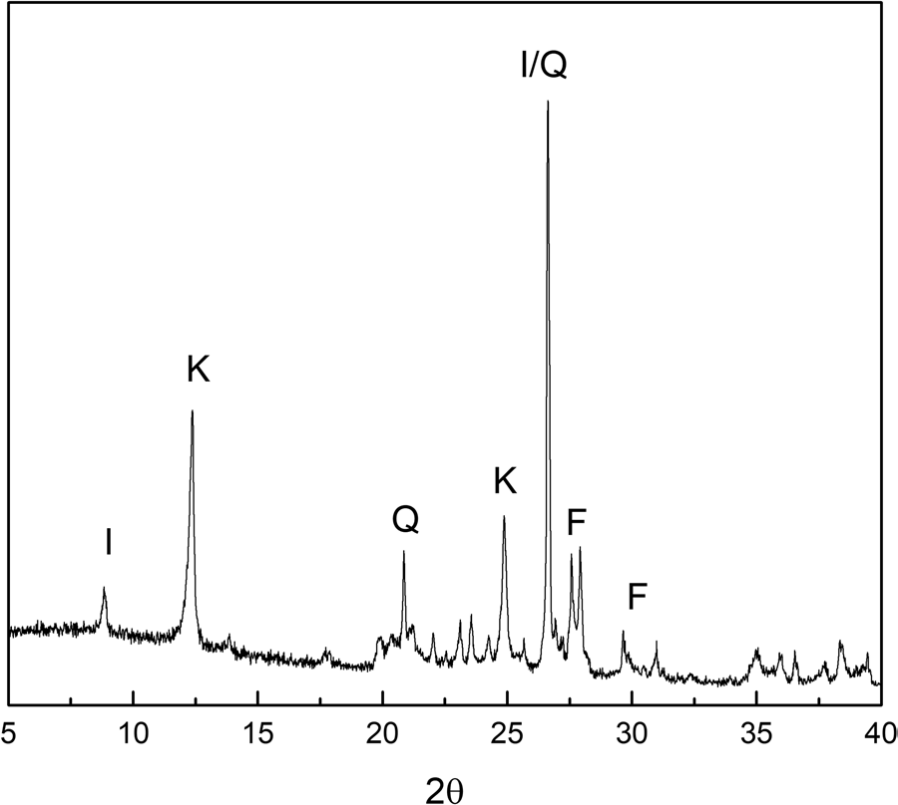
XRD pattern of the Limoges porcelain (I = illite; K = kaolinite; Q = quartz; F = sodium/potassium felspar minerals).

Porcelain is the pinnacle of ceramic production for its exceptional compactness. The density is between 2.2 and 2.6 g/cm^3^, the compressive strength is around 5000 kg/cm^2^, while the tensile strength around 350, the elastic modulus is 800000 kg/cm^2^ and the open porosity, expressed as quantity of absorbed water, does not exceed 0.1%. For this reason, the porcelains are waterproof, unstained, show a high resistance to crushing (2–2.3 kg/cm^2^), abrasion (the Mohs hardness is around 7), scratch, impact, chemical agents with the exception of hydrofluoric acid and very basic hot solutions, they are good conductors of heat and resist well to its action, they have a low electrical conductivity even under very severe conditions^21^. In the present study a Limoges porcelain (supplied by Imerys) was used. The porcelain mixture presents kaolinite and illite as plastic components, together with sodium/potassium feldspars, nepheline and quartz (Fig. 6).

The selected ceramic pigment (0.1 A/900) was added into the porcelain paste in ratio of 10% by weight. The pastes were shaped, dried and, since the thermal treatments were performed in an oxidant atmosphere, fired at 1240 °C.

The typical thermal cycles used in the porcelain specimen manufacture at Capodimonte factory were reported in Supporting Information.

**Figure 7:**
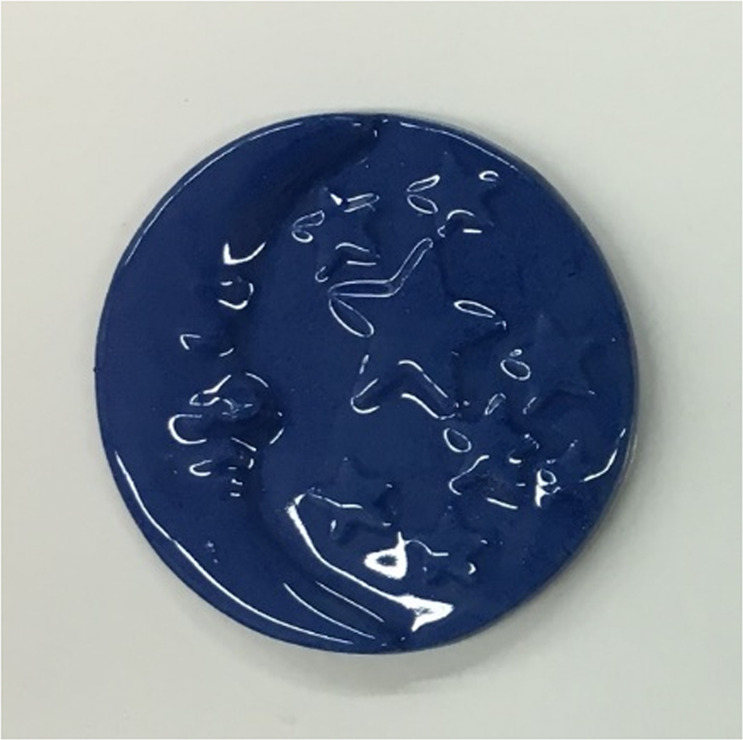
Coloured porcelain body obtained with 10% of zeolite based cobalt pigment.

As showed in Fig. 7 (samples loaded with 10% of pigment), the zeolite-based cobalt pigment was stable at the firing temperature of porcelain mixture and its presence in the mixture did not affect the final product. Moreover, the colour centres seem to be homogeneously dispersed in the porcelain matrix.

The zeolite-based ceramic pigments were also tested as colouring agent in the decoration step. There are a lot of methods to decorate a porcelain product: one of the most common is *ingobbio*^24,25^. It is a layer of clay, white or colored, which covers the object (an enamel can cover it to give shine to the product). The *ingobbio* was obtained by mixing fixed amount (5% by weight) of pigment in the porcelain powder. Then it was applied to the porcelain product by brushing or by casting.

It is also possible to add colour to the glaze to obtain a glossy and coloured coating. The glass coatings are made of fusible glass of different composition and make the substrate impermeable and more aesthetically pleasant. Different percentage of pigments (5 or 7% by weight) were dispersed into the glaze. The glazing was carried out by immersion of the fired samples.

Porcelain is an object of great artistic value, so it is often decorated by hand. The decoration can be performed at different stages of the production process. The pigment, before application on different supports, has been dissolved in painting oil. This technique is defined “third fire decoration” and it is performed on finished products, glazed or not, but they have already undergone the second firing. In fact, the decoration is stabilized at significantly lower temperatures, 760° C, and with a very fast thermal cycle.

The samples were decorated at *third fire* by brush on different supports (with or without glazing). For this decorative technique it is necessary to add to the pigment a low-melting substance in order to favor the adhesion of the decoration on the substrate, since the stabilization of the color takes place at low temperature, on an already consolidated product, so there is no formation of vitreous phases in the matrix that favor the absorption of the decoration^25^.

Some porcelain specimens were decorated by “under cover decoration”. This method is applied on product after the first firing, so the stabilization of the decoration occurred with the second firing. This technique gives higher protection to the décor, since it is cover by glazing and is not in contact with external agents (as food or detergents).

A different aesthetic effect can be achieved without glazing step by “great fire decoration”, decoring the sample by brush or by sponge.

Table 3 summarized all the decoration techniques with process details and images of the resulted products before firing.

**Table 3:** Summary of the tested decoration technique.

Decoration technique	Phase	Stabilization temperature, °C	Amount, %	Aspect of the products
*Ingobbio*	Brushing	900	5	
	Casting	“	“	
Coloured glazing	After first firing	1240	5	
	“	“	7	
Third fire decoration	After second firing	760	-	
Under cover decoration	After first firing	1240	-	
Great fire decoration	After first firing	1240	-	

**Figure 8:**
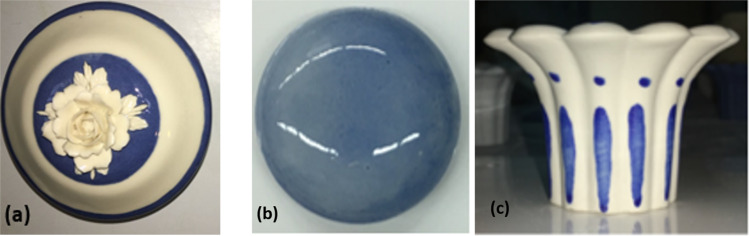
Porcelain products after firing with: (a) ingobbio; (b) glazed and (c) hand decoration.

The application of the zeolite-based pigment in the *ingobbio* allowed to obtain a particular *nuance*, such as Sevres blue, a typical colour of porcelain decors (Fig. 8a). The pigment was added in the glazing to obtain a coloured and glossy coating. The presence of the pigment does not affect the adhesive, gloss and fluidity properties of the coating, but some colour centres were visible after burning. This means that a harder grinding is required before mixing pigment with glaze (Fig. 8b). Finally, the pigment was used to decorate by hand the porcelain objects. Depending of the *décoring* stabilization temperature, a different colours nuance was obtained (Fig. 8c).

## Conclusions

Collected data reveal that the ion exchange mechanism allows to control, at microscopic level, the composition of the zeolitic precursor. In fact, starting from the same support, the colour tone of the final product could be firstly chosen by selecting chromophore cation in the contact solution. Moreover, several chromatic effects could obtain by varying either the cation concentration or the treatment temperature. The resulting pigment can be defined as nanostructured, in fact the colour centres are homogeneously distributed in the support at the atomic level, due to the ion exchange mechanism. This involves in a high colour rendering of the final product. Compared to traditional pigments used in the ceramic industry, the novel zeolitic based pigments appear highly competitive from an environmental and economic point of view.

All the pigments showed a good thermal stability in the typical firing temperature range of porcelain (1240–1280 °C depending on furnace atmosphere is oxidizing or reducing). Good results were obtained in the porcelain manufacture process, in terms of colour rendering and pigment stability.

## Supplementary information

Supplementary information.

## Data Availability

The datasets generated during and/or analysed during the current study are available from the corresponding author on reasonable request.
