# Supplementary material for: Facile synthesis of nanostructured cobalt pigments by Co- A zeolite thermal conversion and its application in porcelain manufacture
Source: Sci Rep. 2020 Jun 23;10:10147. doi: 10.1038/s41598-020-67282-1 (PMC7311435; doi:10.1038/s41598-020-67282-1)
Supplement: Supplementary file 1 — Supplementary information. [file 41598_2020_67282_MOESM1_ESM.docx]

**SUPPORTING INFORMATION**

**Facile synthesis of nanostructured cobalt pigments by Co- A zeolite thermal conversion and its application in porcelain manufacture^§^**

*Assunta Campanile^1^, Barbara Liguori^1,2*^, Ottavio Marino^1^, Gennaro Cavaliere^3^, Valter Luca De Bartolomeis^3^, Domenico Caputo^1,2^*

^1^ACLabs – Dipartimento di Ingegneria Chimica, dei Materiali e della Produzione Industriale Università di Napoli Federico II, P.le V. Tecchio 80, 80125 Napoli, Italia

^2^ INSTM - Consorzio Interuniversitario Nazionale per la Scienza e Tecnologia dei Materiali, Via G. Giusti 9, 50121 Firenze, Italia

^3^ Istituto di Istruzione Superiore ad Indirizzo Raro "Caselli - De Sanctis" di Napoli, Parco di Capodimonte, 80131 Napoli, Italia


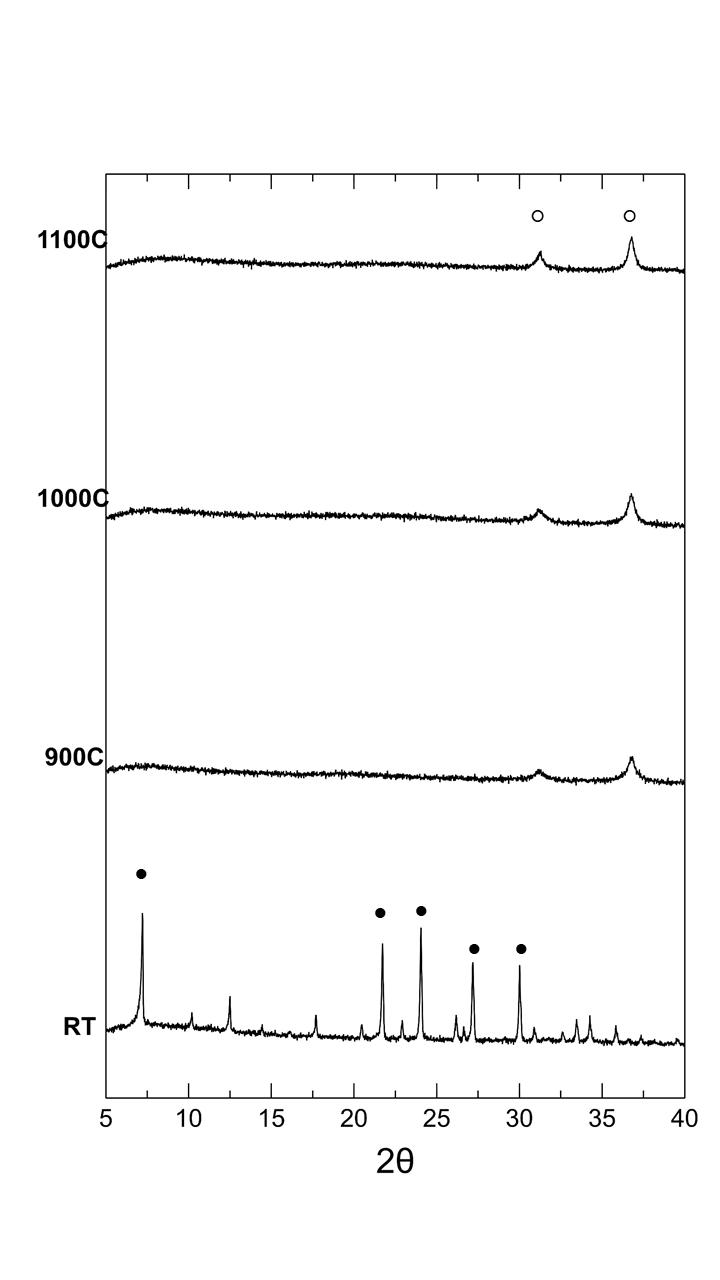


SI_1. XRD patterns of the 0.15-sample treated at different temperatures (●: Co/Na-A; ○: cobalt aluminate)


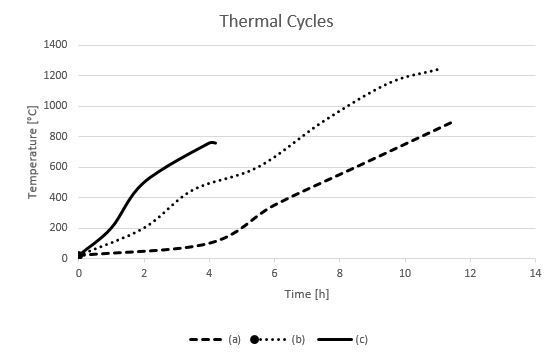


SI_2. Thermal cycles for (a) the first, (b) the second and the third (c) step of the porcelain manufacture
